# Supplementary material for: Cis-activation in the Notch signaling pathway
Source: eLife. 2019 Jan 10;8:e37880. doi: 10.7554/eLife.37880 (PMC6345567; doi:10.7554/eLife.37880)
Supplement: Figure 5—source data 1. [file elife-37880-fig5-data1.zip › Figure 5 - source data 1/README.rtf]

# 2018-05-01# Nagarajan Nandagopal, Leah Santat, Michael ElowitzRunning 'quickrun.m' will generate plots contained in panels D and E of Figure 5, and the distribution of parameters shown in Figure 5 - figure supplement 1. Plots are based on simulation data contained in the 'Simulations' folder. 'extract_solution_features.m' and 'find_successful_combos.m' are used to calculate degree of non-monotonicity for each model and parameter set and number of non-monotonic parameter sets, respectivelys. The Simulations folder contains the models for single (Model 0) and double (Models 1 and 2a-d) cis-complexes in the 'single_complex.m' and 'double_complex.m' files, respectively. Parameter scans were accomplished using 'scan_single_params.m' and 'scan_params.m' respectively. The results of parameter scans are contained in the .mat files.  
